# Additive manufacturing of microplastic reference materials through microextrusion provides monodisperse and exactly counted particles

**DOI:** 10.1038/s41598-025-29499-w

**Published:** 2025-11-26

**Authors:** Maurice Hauffe, Lucas Kurzweg, Robert Möhn, Tilmann Priebe, Thomas Himmer, Arne Cierjacks, Kathrin Harre

**Affiliations:** 1 Faculty of Mechanical Engineering, Hochschule für Technik und Wirtschaft Dresden – University of Applied Sciences, Friedrich-List-Platz 1, 01069 Dresden, Germany; 2 Faculty Agriculture/Environment/Chemistry, Hochschule für Technik und Wirtschaft Dresden – University of Applied Sciences, Friedrich-List-Platz 1, 01069 Dresden, Germany; 3 Physical Chemistry and Polymer Physics, Leibniz-Institute of Polymer Research Dresden e.V., Hohe Straße 6, 01069 Dresden, Germany

**Keywords:** Microplastics, Reference materials, Plastic crisis, Novel entities, Environmental monitoring, Environmental monitoring, Environmental sciences, Materials science, Analytical chemistry, Materials chemistry, Polymer chemistry

## Abstract

Reference materials play a fundamental role in standardizing and validating analytical and technical processes in research and industry. This also applies for the analysis of microplastic pollution, which should allow for both particle count and weight assessment. Here, we showcase a novel production method for monodisperse and count-accurate microplastic reference materials (MRM) using microextrusion via adapted nozzles in commercially available 3D-printers. The method is highly reliable and produces approximately 1,000 particles h^− 1^ with defined size and shape. We explored different thermoplastics such as LDPE, PA, PLA, PCL, and PMMA and size classes (224 –1,349 μm in diameter) concerning size and shape accuracy and reproducibility. The resulting particles showed low variability in size with a standard deviation between 3 and 30%. Particle size was adjusted using different nozzle diameters. Our approach provides a wide range of possible MRM readily available for analysis, process validation and environmental monitoring of microplastics. These reference materials may significantly contribute to the understanding of behaviour and dynamics of microplastics as novel entities with potentially detrimental effects on environment and human health.

**Supplementary Information:**

The online version contains supplementary material available at 10.1038/s41598-025-29499-w.

## Introduction

The availability of accurate, monodisperse microplastic reference materials (MRM) is essential to standardize and validate analytical techniques and technical processes in research and industry. Microplastics (MP) are particles consisting of synthetic polymers that are smaller than 5 mm in size^1^. It has widely been reported that MP are distributed in all environmental compartments around the globe^2^. Consequently, there is high demand for analyzing MP from environmental samples^3–10^. In recent years, MP analysis has made significant progress, which may not only be related to public pressure and growing environmental awareness but also to legal requirements. The EU Directive (EU) 2020/2184 requires all EU member states to implement methods for measuring MP content in drinking water^11^. Also the EU Urban Waste Water Directive, recently amended in 2024, limits the content of MP in wastewater and regulates avoidance strategies^12^.

One of the main challenges in microplastic monitoring is the lack of standardized and cost-effective analysis protocols. Possible methods comprise mass-determining methods such as Pyro-GC/MS, TED-GC/MS and DSC^13–17^ and particle-counting methods such as visual identification along with FTIR and Raman microscopy^4,18^. Unfortunately, both analysis techniques produce data that are not comparable with each other. This was evident in e.g. two MP studies on Elbe river sediments^6,19^ from partly identical sample sites but with different analysis techniques. To overcome these limitations, standard particles are needed that are characterized by a monodisperse size distribution and defined number and shape, as highlighted by several studies^19–21^. Until now, different methods for MRM production have been proposed^19,22,23^.

One method uses cryogenic grinding and screen fractionation, but the use of liquid nitrogen and the fact that this method results only in dispersed fractions with limited size control leads to high energy and material consumption^23^. Furthermore, it is not possible to produce a defined count of particles, nor can it be estimated due to varying particle sizes. The determination of the exact particle count in MRM is based on counting, which is very time-consuming, expensive and error-prone^19,23^. Cryogenic grinding and screen fractionation can neither be used to produce a defined particle size and in most cases, a large quantity of particles with unneeded sizes is obtained in addition to the target fraction^24–26^. Synthetic production of MRM faces similar problems with size distribution and unknown particle counts and is additionally limited to a few polymer types^27,28^. Novel approaches for count-accurate MRM^19,29^ also are available for only one or very few polymers or generate a lot of waste. All these constraints highlight the need for improved monodisperse and count-accurate MRM manufacturing methods.

In this study, we explore a new approach that enables the production of monodisperse MP particles through additive manufacturing, offering a faster and simple solution with the potential of complete automation and inline process control. The method uses 3D-printers that manufacture MP particles via microextrusion with different adapted nozzles^30^. The MP particles can be produced in exact counts as well as in more or less homogeneous size and shape. Therefore, our method makes it possible to compare mass-based and count-based analysis methods for the first time. The study aims at testing the accuracy in count, size, and shape of different thermoplastic polymers such as LDPE, PA, PLA, PCL, and PMMA. Based on this data, the potential for future application of the MRM will be discussed.

## Materials and methods

### Polymer filaments

Both commercially available and self-extruded polymer filaments were used. The key information about these filaments is shown in the supplementary information **ST 1**. The filaments from PLA (PLA + white – eSUN), fluorescent PLA (PLA glow green – eSUN), PCL (Facilan PCL 100 – 3D4Makers), PMMA (PMMA transparent – Material4Print) and PA (PA6/66 (Nylon) – Flashforge) were used as purchased. The LDPE filament was produced on a Collin Teach Line CE20 extruder in combination with the water bath WB850 and the filament winding unit BAW130 from LDPE granulate (LyondellBasell, Lupolen 2420 K). The parameters for filament manufacturing are given in the supplementary information **ST 2**. During production, a diameter of 1.75 mm of the filament strand was continuously checked using an inline filament diameter measuring device. The filaments were stored in a silica-filled airtight box with relative humidity of 10%.

### Characterization of polymer filaments

Information on the commercially purchased filaments is provided by the manufacturers in available data sheets. Self-extruded LDPE filament was characterized through differential scanning calorimetry (DSC) measurement, microscopy, and the measurement of filament diameter.

DSC measurement was carried out using the DSC 214 Polyma from Netzsch. The temperature program was structured as in Schirrmeister et al.^17^ as follows: (1) Heating from room temperature to 300 °C; (2) Isothermal phase for 5 min; (3) Cooling down to – 50 °C at a cooling rate of 20 K min^− 1^; (4) Isothermal phase for 5 min; (5) Heating up to 300 °C at a heating rate of 20 K min^− 1^; (6) Cooling down to room temperature. While measuring, the oven of DSC was purged with argon (60 µl min^− 1^). Polymer samples of 1 g were weighted into 40 µl aluminum crucibles from Netzsch and sealed with a pierced aluminum lid.

For the observation and assessment of filament morphology, the microscope DM2500 M from Leica with the built-in camera Digicam DFC290 (Leica) and the software Leica Application Suite V3.1.0 was used.

Filament diameter was recorded using calliper measuring two times (crosswise at an angle of 90°) along the filament strain at 14 measurement points (every 10 m). Based on this data, average diameter and ovality were determined. The ovality was calculated using the following formula according to DIN EN 10253-2:

1 $$\:{O}_{V}=100\:\%\:\bullet\:\frac{\left({D}_{max}-{D}_{min}\right)}{D}\:\:\:\:in\:\%$$

### Nozzles and printers

For microextrusion, the CNC-controlled cartesian FLM 3D-printers Neptune 4 Pro from Elegoo were modified. Conversions and modifications included hardware and software adjustments to increase precision and accuracy of the device and to produce micro-particles from polymer filaments. A general schematic design of the 3D-printer and the hot-end and extruder unit along with detailed information on the modifications of the 3D-printers can be found in the supplementary information **SF 1** and **SF 2**, respectively.

We used standard brass nozzles with opening diameters of 0.1 mm, 0.2 mm, and 0.4 mm, which were purchased from manufacturer Brozzle. Additionally, brass nozzles with an opening diameter of 0.08 mm were custom-made by micromachining with lathes and laser drilling. Specific parameters can be requested from the authors.

Extrusion trials were carried out as initial tests for nozzles and polymer filaments. Thereby, the speed at which the filament is fed to the nozzle and the extrusion temperature of the nozzle were varied until a polymer strand was constantly extruded with minimum extrudate swell. The particles were directly applied to a printing plate with an area of 235 mm × 235 mm consisting of uncoated steel. 210 mm × 210 mm of the printing plate were used as area for the particle printing. For particle production with the PMMA filament, 3DLAC from the reseller Laboratorios 3D Print S.L. had to be used as adhesion promoter.

### Additive manufacturing of microplastic particles

The modified 3D-printers were controlled with a CNC machine code (G-code) to specify the temperatures of nozzle and printing plate, the feed distance (distance by which the filament is pushed forward through the extruder and thus extruded through the nozzle), and the filament retract along with the targeted number and distance of particles. An overview of the adjusted parameters is given in the supplementary information **SF 3**. To protect the polymer filaments from dust and moisture during printing, the filament spool was located in an actively heated drying box (temperature: 50 °C). The printer was also located in a self-built dust-tight enclosure.

Before each print, the printing plates were cleaned with ethanol and dust free tissues. Particles were produced by feeding the filament to the heated nozzle, subsequent melting, setting down the drop on the printing plate and cooling down to the final particle. To prevent further polymer melt from dropping out, the filament was retracted with a defined distance and speed to create a vacuum at the nozzle opening. The key parameters for the various nozzles and filaments used are given in Table 1.

**Fig. 1:**
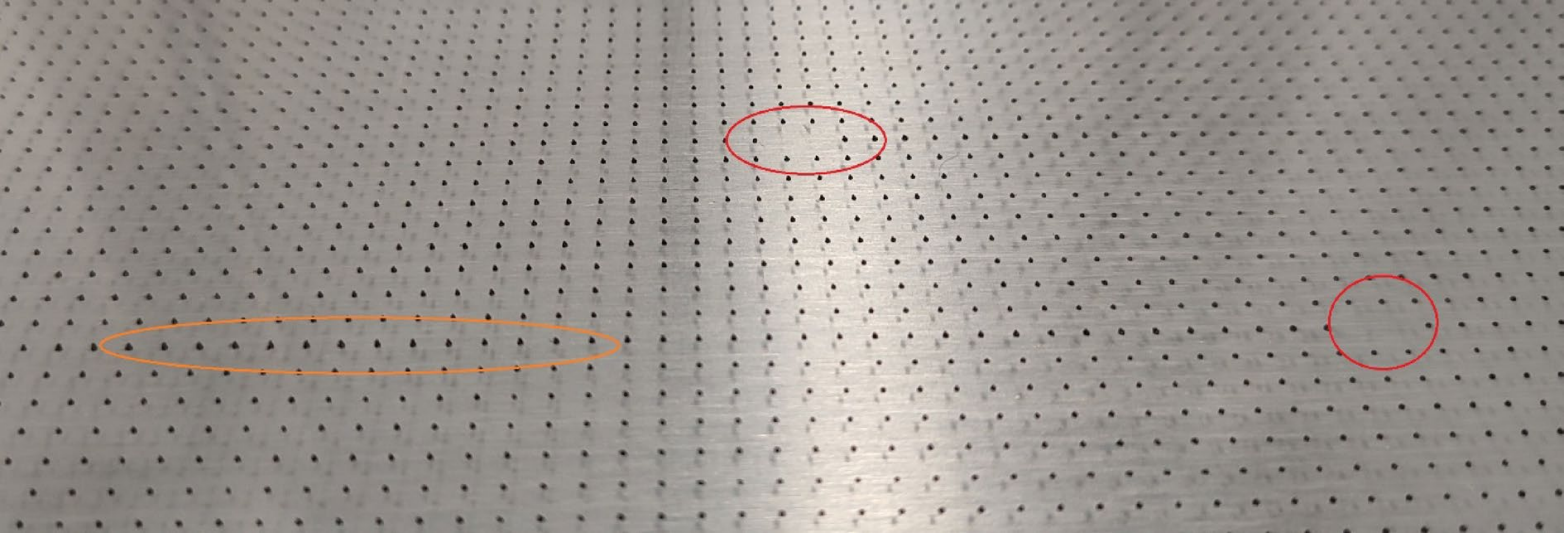
Printing plate with particles made of black PLA with missing particles (red circles in the back and right) and a few over extruded particles (orange oval left).

After printing the particles, we visually inspected the printing plate for missing particles and homogeneous particle sizes as shown in Fig. 1. Due to inherent particle inhomogeneity during the first printing phase of each new printing process, the first 100 particles per printing plate were discarded. The remaining particles were wetted with ethanol (water in case of PMMA) and carefully removed from the print plate using a sharp blade and subsequently transferred into a glass snap lid jar. Particles were dried in an oven at 60 °C. PMMA particles were, before drying, placed for 20 min in deionized water, rinsed 5 times with fresh deionized water and vacuum-filtrated.

**Table 1:** Key parameters of the additive manufacturing of MP particles.

Filament	Nozzle diameter in µm	Nozzle temperature in °C	Bed temperature in °C	Filament feed distance in mm	Feed speed in mm/s	Retract distance in mm	Adhesion promoter
LDPE	400	270	80	0.15	1	3	None
	200	290	80	0.01	0.42	1	None
	100	290	110	0.001	0.25	1	None
PA	200	270	110	0.025	1	2.5	None
	100	280	110	0.5	0.5	6	None
PLA	400	185	110	0.1	5	5	None
	200	185	110	0.015	2	5	None
	100	190	110	0.05	1	5	None
	80	190	110	0.01	1	5	None
PLA glow	400	230	65	0.085	2	2.5	None
	200	230	65	0.01	1	2	None
PCL	400	120	90	0.12	0.5	5	None
	200	150	90	0.08	0.2	5	None
PMMA	200	230	110	0.08	0.2	3.5	3DLAC

### Characterization of the manufactured particles

The characterization of diameter, roundness, and the monodispersity of particle size was carried out after removal, washing and drying. 10% or at least 10 particles from the whole fraction were taken for characterization to align with the guideline ISO 33405:2024. Particle sizes of the manufactured particle samples were determined by microscopy using a DM2500 M from Leica with a Digicam DFC290 and Leica Application Suite V3.1.0 at a magnification of five. For the particle size the diameter of the planar base of the particles was determined. The accuracy of the microscope’s measuring function was regularly checked for deviations. Particle diameters, maximum and minimum Feret diameters, and shape parameters (roundness and aspect ratio) were measured automatically using the software ImageJ (version: 1.54 g, Java 1.8.0_345 64-bit^31)^. The implemented measuring function was calibrated with the scale bar from the Leica software. Average particle diameter was calculated from the mean value of maximum and minimum Feret diameter according to Formula 2.

2 $$\overline{\varvec{X}}_{\text{Ferret}}= \frac{ {\varvec{X}}_{\text{Ferret},\,\text{max}}+ {\varvec{X}}_{\text{Ferret},\,\text{min}} }{2}$$

Masses of the particles produced with a 200 μm nozzle were determined by weighting five fractions with *N* = 50 particles each. The precision scale XSR 105 DualRange from Mettler Toledo with an accuracy of ± 0,01 mg was used. IR spectra were recorded for all particles produced with the 200 μm nozzle using the Bruker Tensor 27 FTIR device with ATR crystal (32 scans, **SF 6** – **SF 11**).

## Results

### Self-extruded LDPE filaments

The average diameter of the filament strain was 1.75 mm, with a standard deviation of ± 0.03 mm. Filament ovality was 1.76%. The detailed results are shown in the supplementary information **ST 3**. Microscopic inspection of the filament did not reveal any detectable defects such as air bubbles or visual cracks. The DSC thermogram of the filament shown in Supplementary Information **SF 4** was in line with pure LDPE polymer.

### Production of particles

Particles were successfully manufactured from PLA, fluorescent PLA, LDPE, PA, PCL and PMMA. Several hundred to over a thousand particles were produced within one hour (Table 2). The maximum number of particles that could be produced in this setup was approximately 5,000 per batch. The count accuracy was high as failures on the print plate could be easily detected by visual inspection of the printed particle grid (Fig. 1).

**Table 2:** Number and mass of MRM particles produced within one hour using a 200 μm nozzle.

Filament	Number of particles	Mass of particle fraction m_f_ in mg	Average mass $\overline{\varvec{m}}$ of one particle in mg
LDPE	858	19	0.022
PA	843	57	0.067
PLA	1,936	57	0.029
PLA glow	1,578	47	0.030
PCL	550	12	0.021
PMMA	1,333	113	0.084

The key production parameters identified during preliminary tests are shown in Table 1. Especially with small nozzle opening diameters, the temperature of the nozzle had to be relatively high in contrast to the manufacturer’s specifications (**ST 1**) or the melting range determined by DSC (**SF 4**).

### Particle properties

**Fig. 2:**
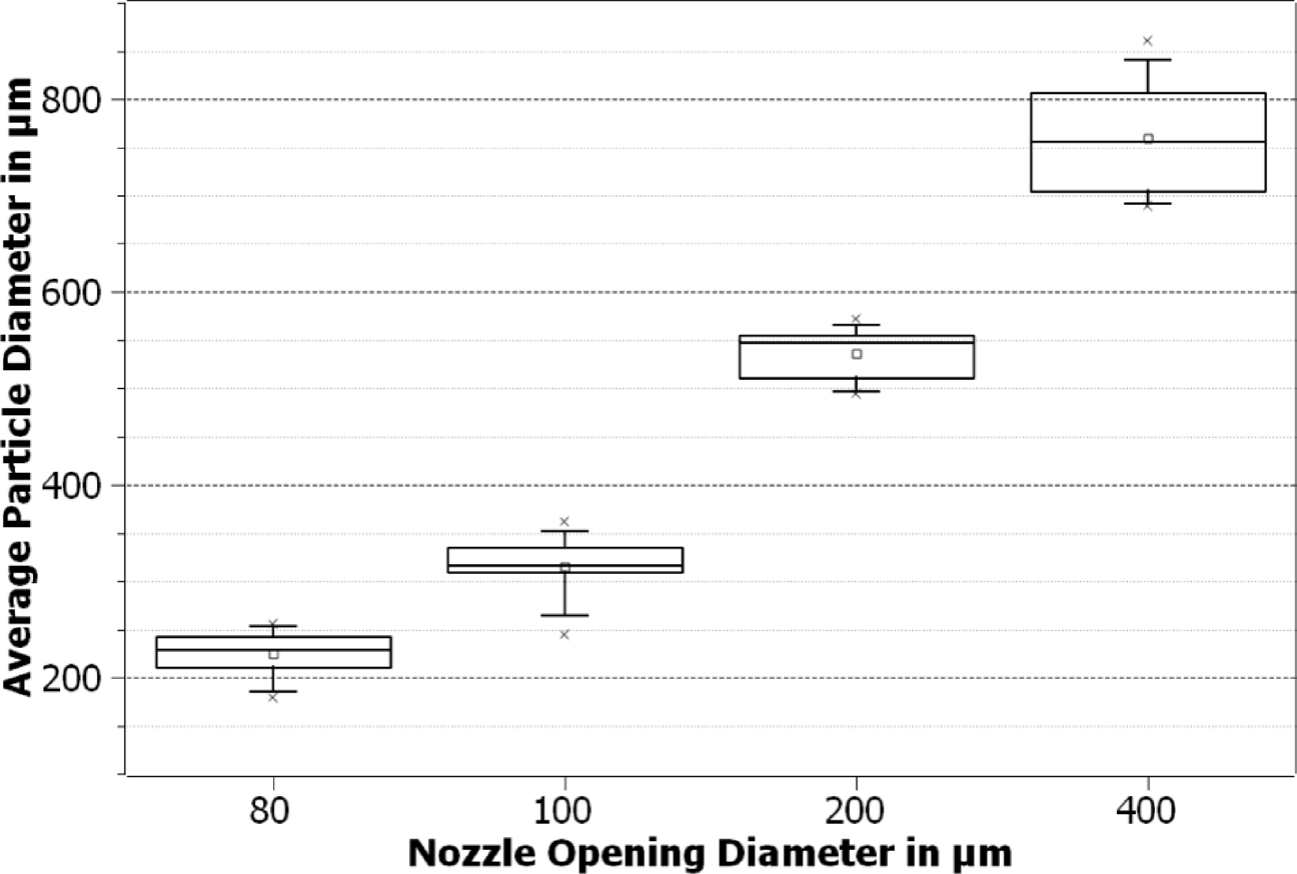
Boxplot of particle sizes for PLA particles manufactured with different nozzle opening diameters.

The tested nozzle diameters enabled precise control of particle size, with smaller diameters allowing for sizes down to 224 μm. All nozzles with opening diameters from 400 μm down to 80 μm were tested with the polymer PLA. The achieved particle sizes of the fractions are shown in Fig. 2. The particle sizes made with the nozzles used varied between around 850 μm and 200 μm.

Particles could be reproducibly manufactured in certain size fractions with a relatively low size variance from LDPE, PA, PLA, PCL and PMMA with average particle diameters between 224 μm and 1,349 μm (as shown in Table 3). The relative standard deviation for almost all fractions was ≤ 15%, except the fraction for PA with the 200 μm nozzle. For the particle fractions with the 400 μm nozzle the standard deviation was < 10% for all tested polymers. The absolute size deviation is below 100 μm, except the particle fractions with the 200 μm nozzle for the polymers PA and PCL.

**Table 3:** Particle sizes for different nozzle diameters and polymers with standard deviation as absolute and percentage value.

Nozzle diameter in µm	Polymer with particle sizes in µm (mean value ± standard deviation, percent standard deviation)					
	LDPE	PA	PLA	PLA glow	PCL	PMMA
400	991 ± 32 (± 3%)	-	759 ± 59 (± 8%)	1,213 ± 67 (± 6%)	1,349 ± 58 (± 4%)	-
200	566 ± 62 (± 11%)	857 ± 256 (± 30%)	536 ± 27 (± 5%)	472 ± 59 (± 13%)	702 ± 105 (± 15%)	902 ± 43 (± 5%)
100	308 ± 26 (± 8%)	575 ± 75 (± 13%)	315 ± 32 (± 10%)	-	-	-
80	-	-	224 ± 25 (± 11%)	-	-	-

**Fig. 3:**
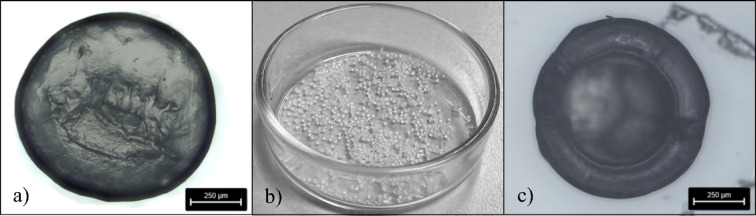
Additively manufactured MRM particles: (**a**) Microscopic image of an LDPE particle produced with a 400 μm nozzle; (**b**) exactly 1,000 LDPE particles in a petri dish produced with a 400 μm nozzle; (**c**) over-extruded PMMA particle manufactured with a 200 μm nozzle.

The shape of the particles can be described either as droplet or hemisphere (shown in the supplementary information **SF 5** and in Fig. 3a). In general, the top view of the particles is round with aspect ratios between 1.04 and 1.84 and values for the roundness of 0.56 to 0.96 (see ST 4).

High count accuracy on a given area along with defined size facilitated exact counting of particles and correlating MRM mass with particle count. For LDPE particles, 1,000 particles were produced within 90 min including set-up and post-processing time of the printing process (Fig. 3b).

Some particles showed signs of overextrusion of polymer, where the polymer melt is squeezed between the print plate and the nozzle (Fig. 3c).

In order to examine the chemical composition of the polymers and rule out possible changes caused by the extrusion process, FTIR spectra were recorded (**SF 6** – **SF 11**). We found no chemical changes in none of the analysed polymers. Spectra confirmed high purity of MRM particles.

## Discussion

Our study demonstrated that microextrusion can be used for manufacturing MRM particles consisting of LDPE, PA, PLA, PCL and PMMA in defined counts and particle diameters.

For most of the polymers and nozzle diameters more than 1,000 particles could be produced within one hour. The maximum number of particles depends on the size of the print plate, the distance between particles and particle size. As the exact particle count is known, the weight of a given fraction can also be used to calculate the mass of an individual particle. The geometric pattern of particles on the printing plate facilitates an easy visual inspection and control of resulting particle counts. (Fig. 1).

Wetting of particles with ethanol or water, respectively, prior to their removal from the printing plate avoided loss of particles due to electrostatic charging or air flow. A further important feature for exact particle number were side walls around the plate holder and a drain through which the particle-liquid suspension could be transferred to a storage container. The accuracy of the counted fractions was verified in several independent tests and no deviation could be detected. As 10% of the total fraction and at least 10 random particles were sampled and analyzed according to ISO 33405:2024, we ensured that a characteristic and representative sample of each particle fraction was analysed.

The study demonstrates that different particle sizes can be manufactured produced by using nozzles with different diameters. However, resulting particle size also depended on the polymer type according to polymer-specific viscosities and rheological behaviour of melts (Table 3). Furthermore, on the production side, errors can result from the used filaments as its accuracy in terms of roundness and diameter is critical for proper extrusion. In the case of purchased filaments, we had to trust in the manufacturers’ specifications (**ST 1**). Still, for the self-extruded LDPE filament, these factors were taken into account (**ST 3**), which resulted in low variation of LDPE particles in comparison to PA particles characterized by clearly higher heterogeneity. The adhesion of the particles to the printing plate may have played an additional role. If the adhesion between the polymer and the printing plate was weak, extrusion rate had to be increased. In the case of PMMA particles, this resulted in a large base area under the nozzle and hence comparatively large particles (Fig. 3c). Consequently, we used an additional adhesion promoter to overcome this constraint. Other polymer types such as LDPE and PLA showed a particularly strong adhesion to the printing plate which enabled the manufacturing of very small particles. In PLA, single particles with a diameter of only 150 μm could be obtained.

For the majority of the particles, a relative standard deviation of the particle sizes of ≤ 10% was observed. According to the definition in VDI guideline 3491, these particle fractions can be considered monodisperse regarding their particle size^32^. In contrast, the particles made of PA, of the polymers LDPE, PLA glow and PCL produced with a 200 μm nozzle, and of PLA produced with an 80 μm nozzle showed higher standard deviations, which should be addressed by future studies.

The use of additively manufactured particles as reference material in microplastic analysis has the potential to significantly enhance the precision and accuracy of this field of study as these particles bridge the gap between particle-counting and mass-determination methods in MP analysis. At present, there is no way to compare these two fundamentally different methods. Using common MP reference materials that consist of cryogenic-milled plastic fragments^22,25,33^ or particles polymerized by direct synthesis^27,28^ makes a mechanistic analysis of the behaviour of MP in the environment inherently difficult. However, the size of the particles is so far for many polymer types too big to model particle movement and sedimentation of MP in a realistic way. Further experiments will be needed to decrease particle size with other manufacturing conditions. Also, particle shape plays a role in interaction with other matrices^29^ and should therefore be considered in future studies.

In contrast to our methodology, the well-established cryogenic grinding process produces a broad particle size distribution that can only be limited by sieve fractionation. Furthermore, producing a specific particle size is not feasible, resulting in a significant portion of particles remaining unused after sieve fractionation^20,24,34^. Depending on the type of polymer and particle size, the target fraction may contain less than 20% of the total amount^26^. In contrast, direct synthesis can produce monodisperse particle fractions, but this method is currently only applicable to limited types of polymers, mainly PS and PMMA^26,27^. Both methods for manufacturing MRM have the disadvantage of being unable to accurately count particles. Manual counting is error-prone and costly in terms of labor, whereas machine counting requires investment in technical facilities^19,23^. Emerging approaches for producing precise MRM comprise the use of CNC milling to selectively remove polymer blocks while preserving structural integrity of individual polymer columns. The resulting void is then filled with gelatine, and thin slices are subsequently cut using a cryo-microtome. Once the gelatine has been dissolved, the resulting solution contains the polymer particles^19^. Again, significant amounts of polymer waste are produced as the quantity of polymer milled from the block exceeds the amount used as particles. Another method uses polystyrene mixed into printer toner to print thin layers onto a water-soluble film with a LaserJet printer. This process enables the precise production of particles with various shapes and sizes^29^. However, exclusively polystyrene has been studied, whereas the feasibility of this approach has yet to be explored with other polymers, and additional post-processing to separate the particles from film matrix is required.

Hence, microextrusion of MRM provides several benefits compared to these methods. In particular, all particles are manufactured in the desired quantity and size at reasonable efforts. Moreover, additive manufacturing of MP particles typically results in minimal polymer waste of just 100 particles per batch, with lower waste-to-yield-ratio being possible when larger printing plates are used. The method is time-efficient and can be easily automated. No manual handling is required during printing process, except for washing PMMA particles. Even manual collection of particles, as described here, may be easily replaced in the future using scraping and extraction devices.

Filaments of nearly all thermoplastic polymers are commercially available, optionally with various additives. Other pure polymer filaments with or without additives can be produced using common extruder systems (here done for LDPE). Extrusion parameters have to be determined through preliminary tests (**ST 1**). For LDPE filaments, temperature tests were based on the melting temperature determined by DSC (**SF 4**). It is important to mention that smaller nozzle diameters require higher extruder temperatures (albeit below decomposition temperatures and taking account Deborah number). MRM particles can be easily distinguished from other particles by their distinctive hemispherical shape with round base area as MP particles found in the environment mostly tend to have an undefined fragment shape^1,2,6,15^. Furthermore, additives such as colorants may be used for better retrieval of MRM particles. Consequently, MRM presented here can be also used as internal or external standard for analytical purposes such as DSC and TED-GC/MS. The defined properties of MRM make them optimal candidates for comparing different analytical methods.

Overall, monodisperse and count-accurate MRM seems particularly promising for studying particle size and shape effects under lab and environmental conditions in food chains, organisms, and ecosystem compartments (soil, water, and biosphere). As such, the development of this straight-forward method for manufacturing reference material will contribute to improve the analysis, the mechanistic understanding, and the management of MP pollution. The proper explanation of the dynamics of MP, as novel entities with impact on nearly all planetary boundaries^34^, will be a fundamental requirement for tackling the global plastic crisis.

## Supplementary Information

Below is the link to the electronic supplementary material.

Supplementary Material 1

## Data Availability

The original contributions presented in the study are included in the article/Supplementary Information, further inquiries can be directed to the corresponding author. A Preprint Version can be found at Research Square (https://www.researchsquare.com/)^35^.

## Abbreviations

CNC: Computer numerical control
DSC: Differential scanning calorimetry
FLM: Fused layer modelling
LDPE: Low density polyethylene
MP: Microplastic(s)
MRM: Microplastic reference materials
PA: Polyamide
PCL: Polycaprolactone
PLA: Polylactic acid
PMMA: Polymethyl methacrylate
Pyro-GC/MS: Pyrolysis-gas chromatography/mass spectrometry
TED-GC/MS: Thermal extraction desorption-gas chromatography/mass spectrometry
