# Supplementary material for: Additive manufacturing of microplastic reference materials through microextrusion provides monodisperse and exactly counted particles
Source: Sci Rep. 2025 Nov 26;15:42560. doi: 10.1038/s41598-025-29499-w (PMC12663106; doi:10.1038/s41598-025-29499-w)

Additive Manufacturing of Microplastic Reference Materials through Microextrusion Provides Monodisperse and Exactly Counted Particles

Maurice Hauffe[a], Lucas Kurzweg[b,c], Robert Möhn[a], Tilmann Priebe[b], Thomas Himmer[a], Arne Cierjacks[b], Kathrin Harre[b*]

[a] Faculty of Mechanical Engineering, Hochschule für Technik und Wirtschaft Dresden – University of Applied Sciences, Friedrich-List-Platz 1, 01069 Dresden, Germany

[b] Faculty of Agriculture, Environment and Chemistry, Hochschule für Technik und Wirtschaft Dresden – University of Applied Sciences, Friedrich-List-Platz 1, 01069 Dresden, Germany

[c] Leibniz-Institute of Polymer Research Dresden e.V., Physical Chemistry and Polymer Physics, Hohe Str. 6, 01069 Dresden, Germany

*** Correspondence:**

Name: Kathrin Harre

E-Mail: kathrin.harre@htw-dresden.de

Address: University of Applied Sciences Dresden, Friedrich-List-Platz 1, 01069 Dresden, Germany

Supplementary Information

Supplementary Table ST 1: Properties of Polymer Filaments

Polymer	Name	Manufacturer	Diameter ± Deviation in mm	Recommended Nozzle Temper-ature in °C	Speciality/ Additive
PLA	PLA+ weiß	eSUN	1.75 ± 0.03	210 - 230	Tough Material
PLA	Luminous PLA glow green	eSUN	1.75 ± 0.03	190 - 230	Fluorescent
PCL	Facilan PCL100	3D4Makers	1.75 ± n. s.	190 - 170	
PMMA	PMMA transparent	Material4Print	1.75 ± 0.02	240 - 270	
PA6/6.6	PA6/66 (Nylon)	Flashforge	1.75 ± n. s.	220 - 260	Polymer Blend
LDPE	CE20_LDPE-st_cl (Lupolen 2420K)	Self-Extruded, (Granulate by LyondellBasell)	1.75 ± 0.03	n. s.	

Supplementary Table ST 2: Parameters for manufacturing of LDPE Filament on The Collin Teachline (CE20 Extruder + Collin Water Bath WB850 + Collin Filament-Rewinder BAW130)

Granulate	Lupolen 2420 K (Manufacturer: LyondellBasell)
Temperature Extruder<br>Zone 1 (Inlet):<br>Zone 2:<br>Zone 3:<br>Zone 4:<br>Zone 5 (Nozzle):	 35<br>170<br>180<br>185<br>190
Nozzle Opening Diameter in mm	3
Filament Exit Angle in °	45
Speed of Extruder Screw in U/min	28
Pressure on the nozzle in bar	14
temperature water bath in °C	60
Path length of Filament through water bath in m	1,2
Take-Off Speed in m/min	5,55
Winding Torque in Nm	0,55

Supplementary Figure SF 1: Schematic design of a cartesian 3D-printer and it's hot-end and extruder unit. In the final experimental setup, the 3D-printer is in a dust-tight enclosure.

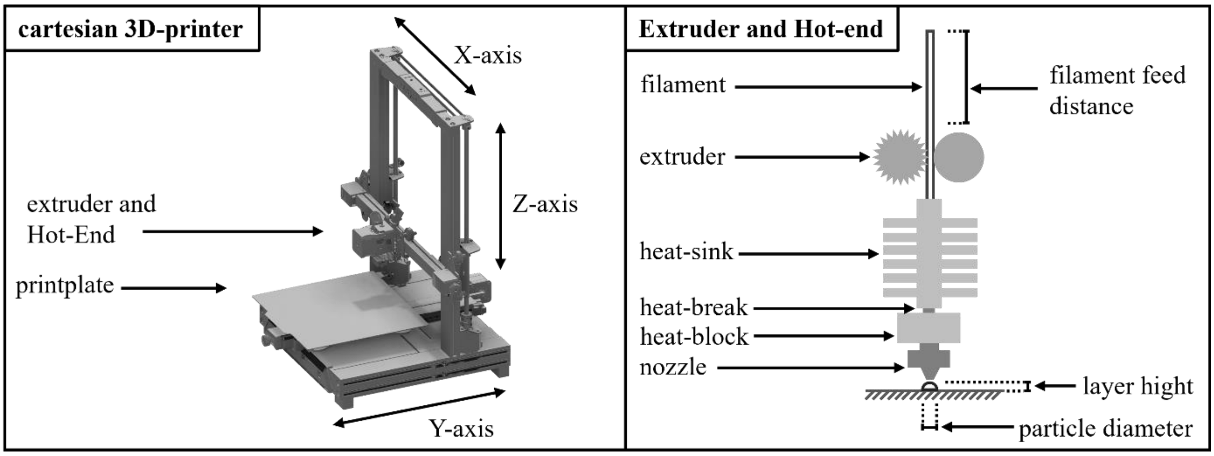

Supplementary Figure SF 2: Modified Elegoo Neptune 4 Pro 3D-printer with more precise z-axis spindels (concealed behind the left and right aluminium extrusions of the printer), exchanged toolhead (exdruder, hotend, measuring sensor for the distance between nozzle and bed), more levelled print bed with glass plate, and optimized stepper motors for all axis and the extruder. Printer is enclosed in dust tight housing out of aluminium extrusions, plexiglass and all is sealed with foam sealing tape. Air purifiers with HEPA filter and activated carbon is inside (black box behind the printer) and on the top (white box on the right) of the housing. The Filament is on the top in dust tight and active heated housing (white box in the middle).

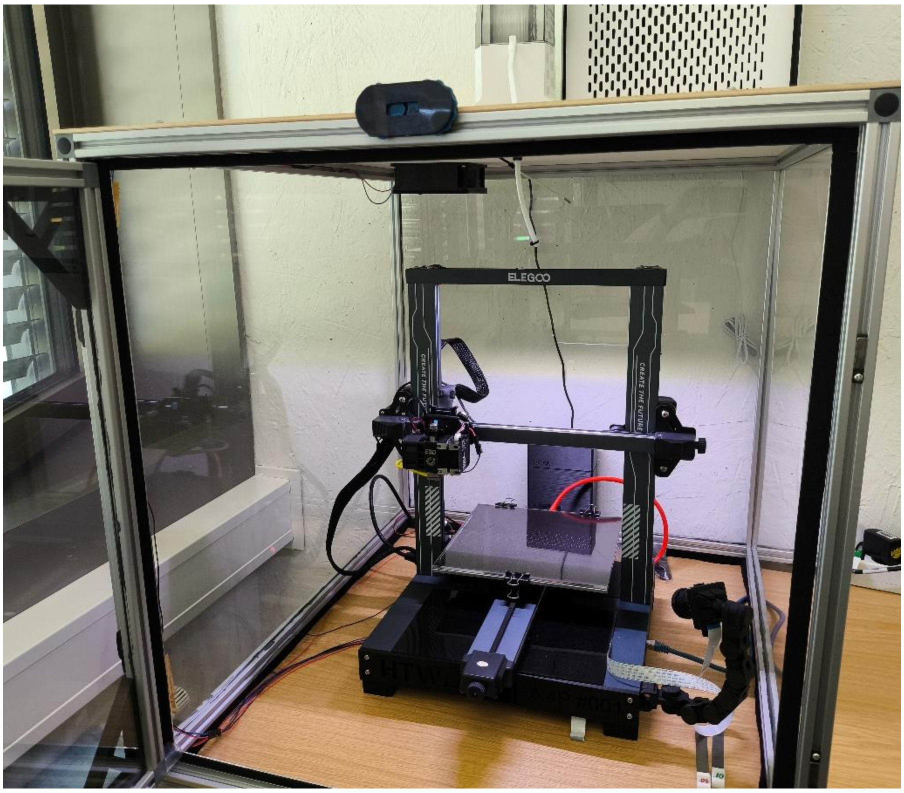

Supplementary Figure SF 3: Screenshot of the selection options in the Python program interface. Print parameters and particle count are stored in the printer-specific and filament-specific configuration files.

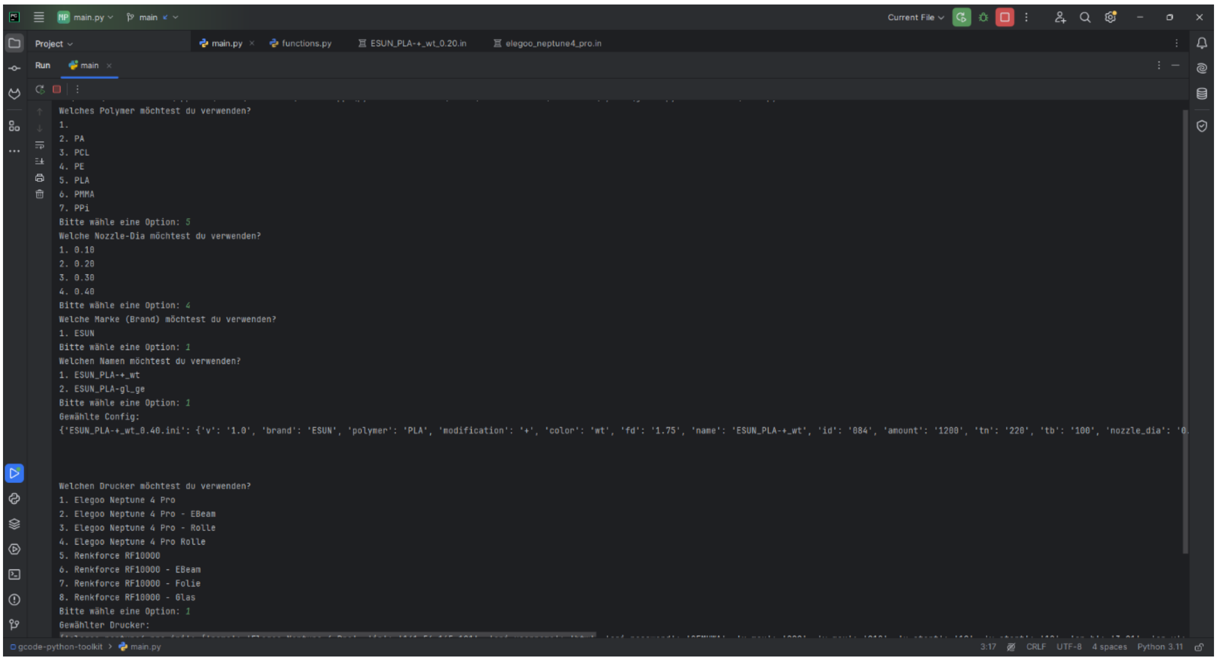

Supplementary Table ST 3: Results of Self-Extruded LDPE-Filament

**Position**	**Diameter 1 in mm**	**Diameter 2 in mm**	**Ovality in %**
1	1,75	1,71	2,29
2	1,75	1,73	1,15
3	1,71	1,75	2,29
4	1,74	1,80	3,44
5	1,78	1,73	2,86
6	1,74	1,77	1,72
7	1,72	1,75	1,72
8	1,72	1,72	0,00
9	1,78	1,78	0,00
10	1,76	1,73	1,72
11	1,75	1,77	1,15
12	1,77	1,71	3,44
13	1,72	1,73	0,57
14	1,74	1,78	2,29
**Average (± standard deviation)**	**1,75 ± 0,03**		**1,76**

Supplementary Figure SF 4: DSC-Measurement of LDPE-Self-Extruded Filament

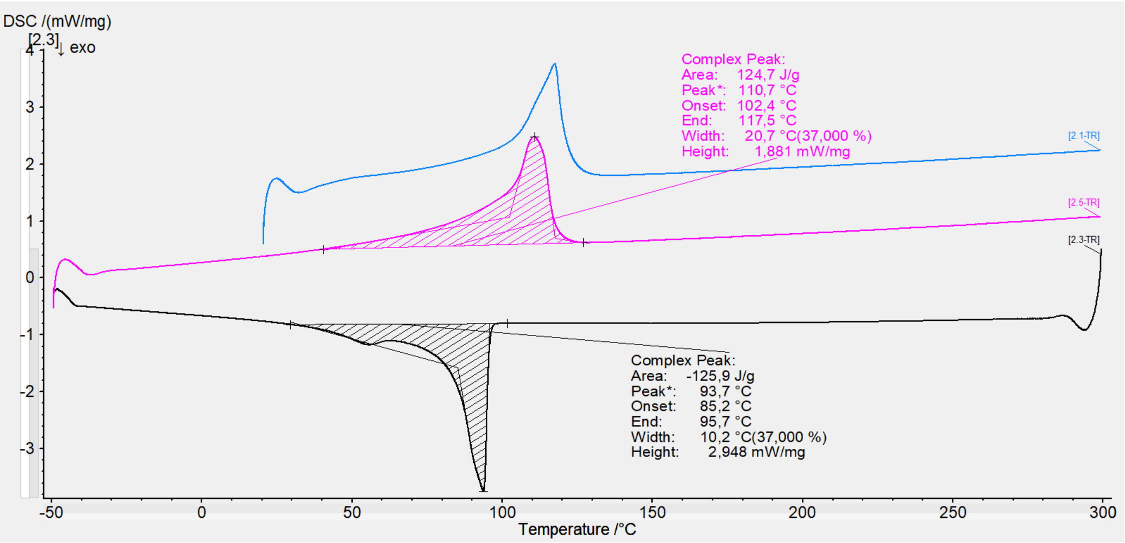

Supplementary Figure SF 5: REM- picture of the Shape of Additive Manufactured Particles from the Side

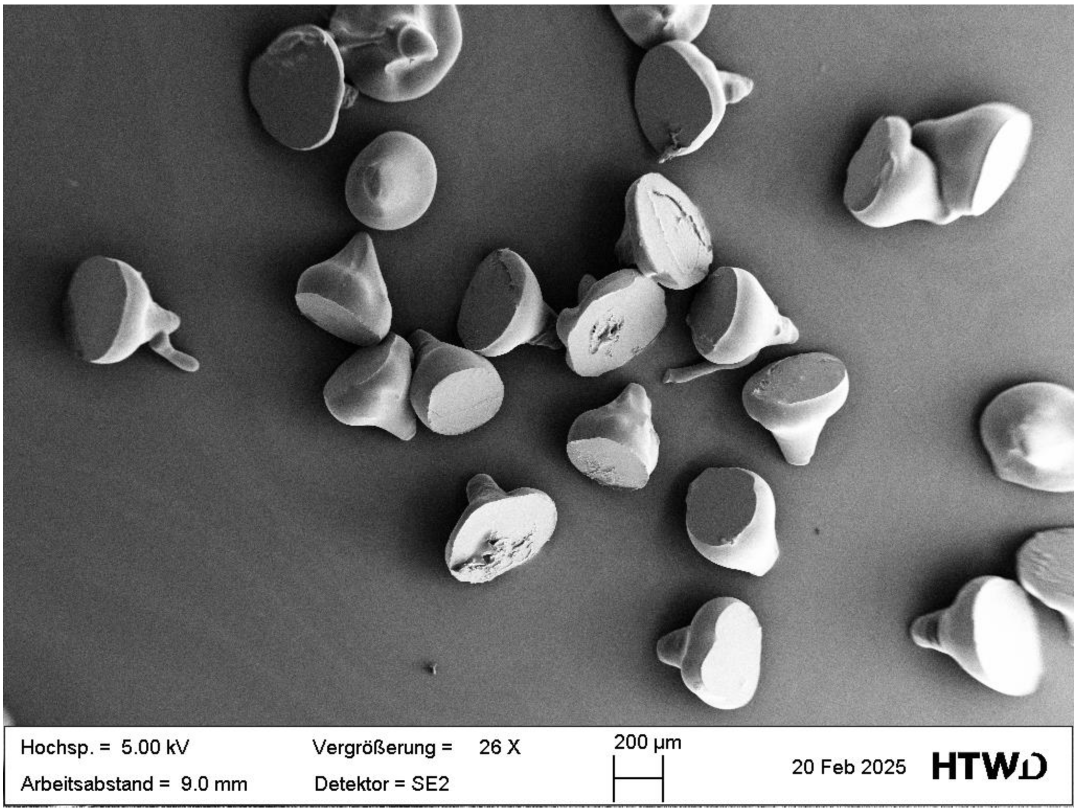

Supplementary Table ST 4: Average Values for Aspect Ratio (AR) and Roundness (R) of the particle fractions

**Nozzle diameter in µm**	**Polymer**											
	**LD-PE**		**PA**		**PLA**		**PLA glow**		**PCL**		**PMMA**	
	AR	R	AR	R	AR	R	AR	R	AR	R	AR	R
**400**	1,07	0,93			1,04	0,96	1,05	0,96	1,57	0,64		
**200**	1,08	0,93	1,84	0,56	1,08	0,93	1,09	0,93	1,10	0,91	1,04	0,96
**100**	1,11	0,91	1,19	0,85	1,42	0,71						
**80**					1,07	0,93						

Supplementary Figure SF 6: ATR-FTIR spectrum from LDPE (extruded with 200 µm Nozzle at 290 °C

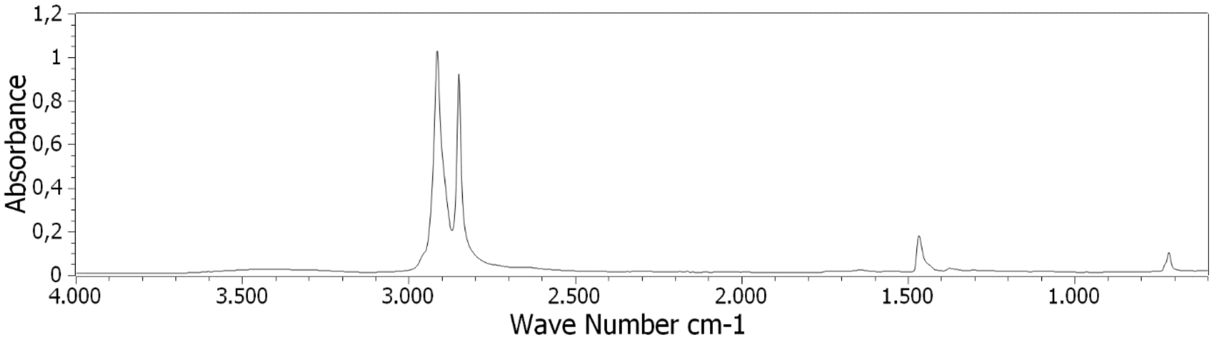

Supplementary Figure SF 7: ATR-FTIR spectrum from PA (extruded with 200 µm Nozzle at 270 °C

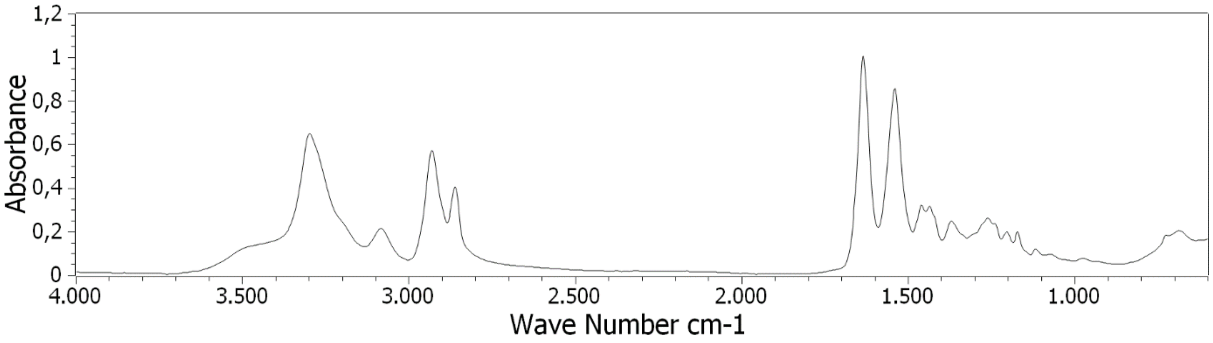

Supplementary Figure SF 8: ATR-FTIR spectrum from PLA (extruded with 200 µm Nozzle at 185 °C

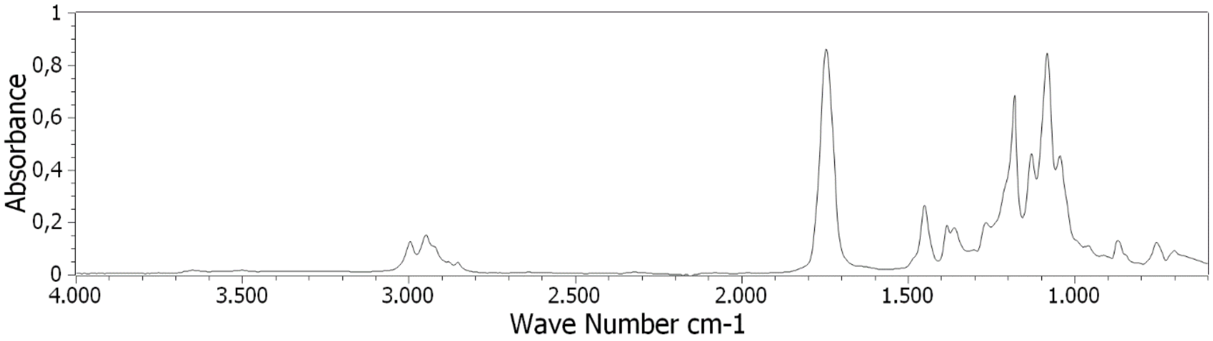

Supplementary Figure SF 9: ATR-FTIR spectrum from PLA glow (extruded with 200 µm Nozzle at 230 °C

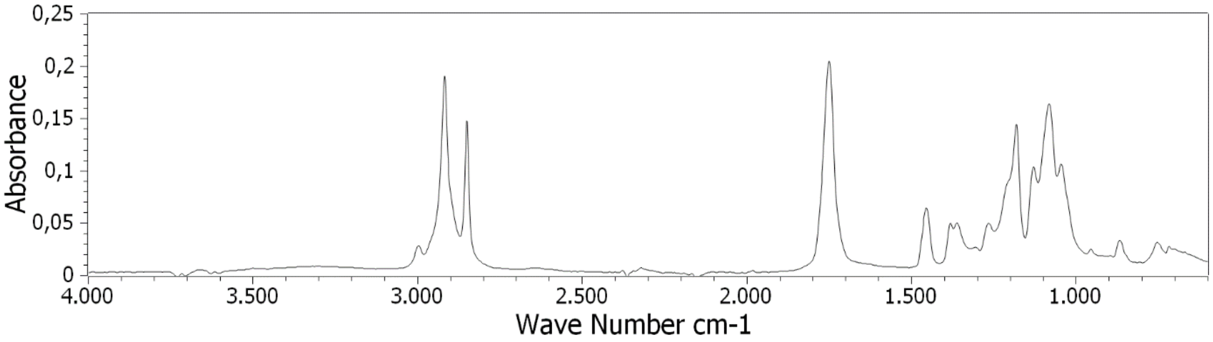

Supplementary Figure SF 10: ATR-FTIR spectrum from PCL (extruded with 200 µm Nozzle at 150 °C

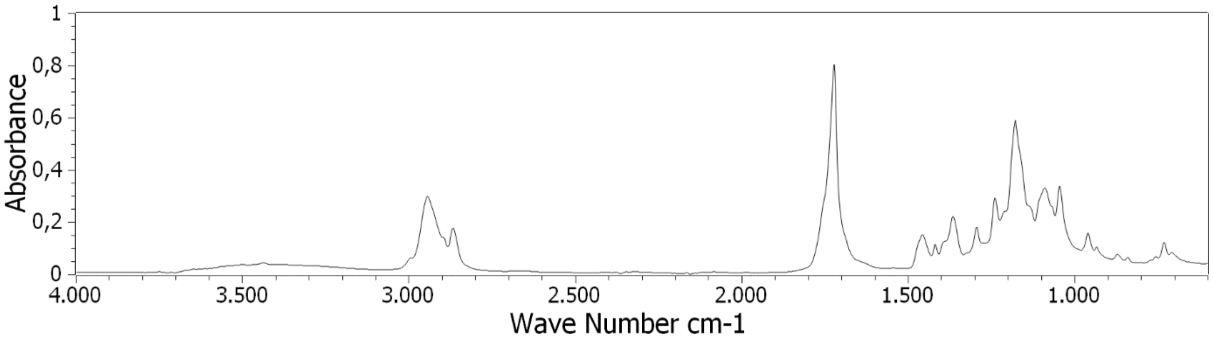

Supplementary Figure SF 11: ATR-FTIR spectrum from PMMA (extruded with 200 µm Nozzle at 230 °C

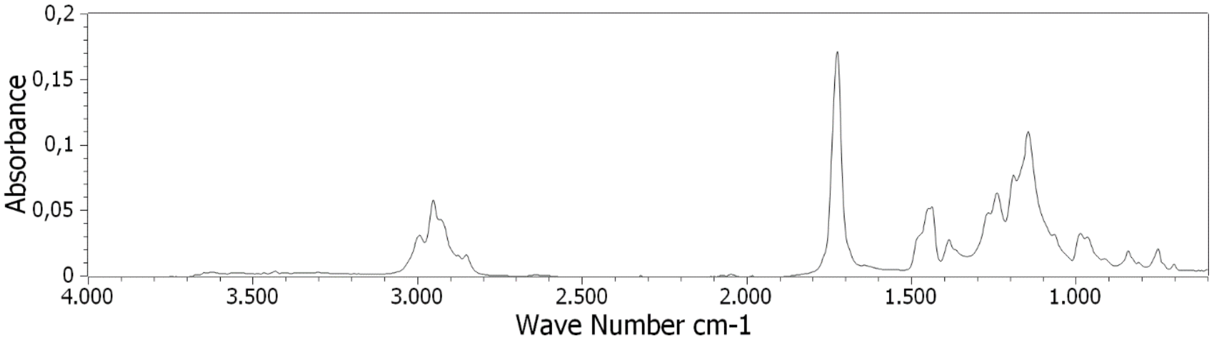

Supplement: Supplementary file 1 — Supplementary Material 1 [file 41598_2025_29499_MOESM1_ESM.pdf]
